# Supplementary material for: Two distinct metacommunities characterize the gut microbiota in Crohn's disease patients
Source: Gigascience. 2017 Jun 26;6(7):1–11. doi: 10.1093/gigascience/gix050 (PMC5624284; doi:10.1093/gigascience/gix050)
Supplement: GIGA-D-17-00073_Revision_2.pdf [file gix050_GIGA-D-17-00073_Revision_2.pdf]

Manuscript

Click here to download Manuscript GIGA-D-17-00073 manuscript_2017-06-01 v2.GY.docx

Two distinct metacommunities characterize the gut microbiota in Crohn's disease patients

Qing He[1,2,3†], Yuan Gao[4,5†], Zhuye Jie[4,5], Xinlei Yu[4,5], Janne Marie Laursen[6], Liang Xiao[4,5], Ying Li[1], Lingling Li[2], Faming Zhang[7], Qiang Feng[4,8], Xiaoping Li[4,5], Jinghong Yu[4,5], Chuan Liu[4,5], Ping Lan[1,3], Ting Yan[2], Xin Liu[4,5], Xun Xu[4,5], Huanming Yang[4,9], Jian Wang[4,9], Lise Madsen[4,10,11], Susanne Brix[6], Jianping Wang[1,3], Karsten Kristiansen[4,10]*, Huijue Jia[4,5,12]*

* To whom correspondence should be addressed: K.K. (kk@bio.ku.dk) or H.J. (jiahuijue@genomics.cn)

† Contributed equally

[1]Department of Gastroenterology, The Sixth Affiliated Hospital of The Sun Yat-sen University, Guangzhou 510610, China

[2]Department of nutrition, The Sixth Affiliated Hospital of Sun Yat-sen University, Guangzhou 510610, China

[3]Guangdong Provincial Key Laboratory of Colorectal and Pelvic Floor Diseases, the Sixth Affiliated Hospital, Sun Yat-sen University, Guangzhou 510610, China

[4]BGI-Shenzhen, Shenzhen 518083, China

[5]China National Genebank-Shenzhen, BGI-Shenzhen, Shenzhen 518083, China

[6]Department of Biotechnology and Biomedicine, Technical University of Denmark (DTU),

Kongens Lyngby, Denmark.

[7]Digestive Endoscopy and Medical Center for Digestive Diseases, the Second Affiliated Hospital of Nanjing Medical University, Nanjing 210011, Jiangsu Province, China

[8]Shenzhen Engineering Laboratory of Detection and Intervention of Human Intestinal Microbiome, BGI-Shenzhen, Shenzhen 518083, China

[9]James D. Watson Institute of Genome Sciences, Hangzhou 310058, China

[10]Laboratory of Genomics and Molecular Biomedicine, Department of Biology, University of Copenhagen, Universitetsparken 13, 2100 Copenhagen, Denmark.

[11]National Institute of Nutrition and Seafood Research, Bergen, Norway.

[12]Shenzhen Key Laboratory of Human Commensal Microorganisms and Health Research, BGI-Shenzhen, Shenzhen 518083, China

Abstract

Background: The inflammatory intestinal disorder Crohn's disease (CD) has become a health challenge worldwide. The gut microbiota closely interacts with the host immune system, but its functional impact in CD is unclear. Except for studies on a small number of CD patients, analyses of the gut microbiota in CD have used 16S rDNA amplicon sequencing. Here we employed metagenomic shotgun sequencing to provide a detailed characterization of the compositional and functional features of the CD microbiota, comprising also unannotated bacteria, and investigated its modulation by exclusive enteral nutrition (EEN).

Results: Based on signature taxa, CD microbiotas clustered into two distinct metacommunities indicating individual variability in CD microbiome structure. Metacommunity-specific functional shifts in CD showed enrichment in producers of the pro-inflammatory hexa-acylated lipopolysaccharide variant and a reduction in the potential to synthesize short chain fatty acids. Disruption of ecological networks was evident in CD, coupled with reduction in growth rates of many bacterial species. Short-term EEN elicited limited impact on the overall composition of the CD microbiota, although functional changes occurred following treatment.

Conclusions: The microbiotas in CD patients can be stratified into two distinct metacommunities with the most severely perturbed metacommunity exhibiting functional potentials that deviate markedly from that of the healthy individuals with possible implication in relation to CD pathogenesis.

Keywords: Crohn’s disease, Gut microbe, Metagenomics, Exclusive enteral nutrition

Background

Crohn's disease (CD) is an inflammatory bowel disease (IBD) that may affect any part of the gastrointestinal (GI) tract. Gut microbes have recently gained much attention as plausible drivers of CD. This notion is supported by the fact that the intimate interaction between the gut microbiota and the intestinal mucosa constantly modulates and shapes the gut immune system[1], and departure from the normal homeostatic microbiome state likely triggers immune dysregulation via pro-inflammatory cues. Specific pathogens that possibly cause CD have been identified, such as adherent-invasive *Escherichia coli* (AIEC) [2] and *Mycobacterium avium paratuberculosis* (MAP) [3]. However, these bacteria were detected only in a fraction of patients [2, 3]. It is therefore assumed that the overall composition of the gut microbiota rather than specific microorganisms accounts for the inflammatory state in CD. Studies using 16S rRNA gene amplicon sequencing to characterize CD-associated microbiota abnormalities revealed an overall reduced microbial diversity in CD [1, 4, 5]. Moreover, a reduction in the relative abundance of *Roseburia* [6], *Faecalibacterium* [1, 5-7], *Bifidobacteriaceae* [1], and *Clostridiales* [5], and an increase in the relative abundance of the *Enterobacteriaceae* family members [1, 4-7] were reported in patients with CD. However, studies using 16S rRNA gene amplicon sequencing have limitations in taxonomic resolution and functional inference. Metagenomic shotgun sequencing can overcome these limitations, but only a few studies have applied metagenomic shotgun sequencing on CD microbiota, including the initial report on 4 CD cases (along with 21 ulcerative colitis cases) to illustrate the utility of the first gut microbial reference gene catalog[8], and a subsequent study on 23

pediatric CD patients[9]. The current incomplete understanding of the functional roles played by the gut microbiota has limited the efforts to devise more targeted treatments.

Conventionally, CD is treated with anti-inflammatory or immunosuppressive medications, or by surgery if symptoms cannot be improved pharmaceutically [10]. However, side effects and complications such as infection and malnutrition accompany these treatments [11], which imperil the patient's life. Although not widely used, exclusive enteral nutrition (EEN) is a low-risk, non-invasive therapy for CD that involves exclusive ingestion of 100% liquid formula made up of either elemental or polymeric nutrients [12]. In pediatric CD up to 85% remission has been achieved by EEN [12]. Nevertheless, in adult CD, EEN has not delivered desirable effectiveness, which to some extent may be attributed to non-adherence and interpersonal variations in clinical conditions [12]. The mechanism underlying the alleviation of CD by EEN also remains unclear, though nutritional improvement and microbial involvement possibly play a role [13]. Although previous studies have described the effects of EEN on the microbiota of pediatric CD[9, 14], it is unclear how EEN modulates their adult counterpart.

Through metagenomic sequencing and data analysis, we herein provide novel insights into the CD microbiota at both compositional and inferred functional levels. We identified two metacommunity stages within CD patients that differed by abundance of gram-negative pro-inflammatory bacteria and presence of genes involved in production of anti-inflammatory short-chain fatty acids. In addition, we investigated the effect of short-term EEN on the CD microbiota. Our study highlights the presence of two microbiota severity-states related to gut

microbiota dysbiosis in CD and indicates possible functional links between the microbiota and the underlying immunological dysbalance in CD.

Data Description

49 CD patients and 54 healthy controls (CTs) were enrolled in this study. 14 CD patients underwent EEN treatment (for the clinical profiles of CD patients, see Supplementary Table 1). Fecal samples were collected from all participants at baseline and from the EEN-treated patients after two-week EEN treatment, totaling 117 fecal samples. After DNA extraction, DNA library of an insert size of 350bp was constructed and then sequenced on an Illumina HiSeq 2000 analyzer at BGI (Shenzhen, China) using 100bp paired-end (PE) sequencing. In total, we generated ~700Gb raw data, and 672Gb of them remained after filtering out low-quality or host reads. The dataset is available from the EBI Database (Accession No. PRJEB15371)[15]. On average ~55.65 million high-quality reads per sample were generated for further analyses. The proportion of high-quality reads among all raw reads from each sample was 95.98% on average. Using both de novo assembly and alignment against the integrated gene catalog (IGC) geneset, 2036584 genes with occurrence rate over 5% were obtained.

Analyses

Clustering of CD microbiota into distinct metacommunities

When the gut microbiotas of CD patients were compared to their non-CD counterparts, both microbial gene counts (**Supplementary Fig. 1a**) and diversity (**Supplementary Fig. 1b**) were considerably lower in CD patients than in CTs. For high-confidence taxonomic identification, co-abundant genes were binned into metagenomics species (MGS) [16] (harboring more than

700 genes) which were thereafter used for taxonomic annotation. A total of 452 MGS were identified, with 151 of them being assigned to existing taxonomic entities (**Supplementary Table 2**).

To capture the principal differences between non-CD and CD microbiome structures, we adopted a combinatory approach which started with sample clustering based on the dirichlet multinomial mixtures (DMM) model [17], followed by the identification of discriminative microbes using an adapted version of the linear discriminant analysis (LDA) effect size (LEfSe) method [18]. Based on Laplace approximation [17], we identified 3 clusters to exhibit minimal negative log posterior (**Supplementary Fig. 1c**). Based on this we clustered the microbiome samples of CD and CTs into 3 metacommunities (A, B and C), which displayed intra-community homogeneity and inter-community dissimilarity (**Fig. 1a**). The membership of a metacommunity was associated with disease status (Fisher's exact test with BH adjustment, $q<0.01$, **Supplementary Table 3**). Metacommunity A was dominated by CT samples and metacommunity C exclusively by CD samples, whereas metacommunity B contained both CT and CD samples (**Fig. 1a**). Based on a less stringent LEfSe method, 85 MGS were identified as discriminative microbes for the metacommunities or sub-groups (CT and CD groups within metacommunity B) (**Fig. 1a** and **Supplementary Table 4**). The majority of metacommunity A-enriched MGS were reduced in metacommunity B and further depleted in C, including short-chain fatty acid (SCFA)-producing bacteria such as *Bifidobacterium* species, *Faecalibacterium prausnitzii*, *Alistipes shahii* and *Roseburia* species (**Fig. 1a** and **Supplementary Table 4**). Among others, SCFA-producing bacteria *Bacteroides*

cellulosilyticus, *Bacteroides xylanisolvens*, and *Clostridium nexile*, a member of the immunomodulatory Clostridium cluster XIVa [19], were enriched in metacommunity B (**Fig. 1a** and **Supplementary Table 4**). Another Clostridium cluster XIVa clade member, *Clostridium symbiosum*, and a number of opportunistic pathogens such as *E. coli*, *Klebsiella pneumoniae*, *Streptococcus salivarius*, and *Clostridium bolteae* were overrepresented in metacommunity C (**Fig. 1a** and **Supplementary Table 4**), suggesting that subjects in this group had impaired ability to suppress colonization by pathogenic species in their gut. We also evaluated whether metacommunities differed in the degree of dysbiosis associated with CD through computing the Microbial Dysbiosis index (MD-index) [5]. CD microbiotas from metacommunity C had significantly higher values of the MD-index than those from metacommunity B (p = 7.63e-05, **Fig. 1a** and **Supplementary Table 1**), suggesting a more severe degree of dysbiosis in this CD subgroup. Combined, these compositionally distinct metacommunities recapitulate disparate configurations of the microbiota under normal and CD conditions.

The separation of microbiomes into metacommunities was confirmed by principal coordinate analysis (PCoA), which clustered samples by both metacommunity identity and disease status (**Fig. 1b**). We determined whether the variations in microbiome composition were accompanied with clinical phenotypes. In CD patients, 23 clinical variables together with age correlated with microbiome variation, with uric acid (UA) and blood leukocyte numbers being the top two covariates (effect size > 0.2) (**Supplementary Fig. 2b**). When categorized into groups, various plasma biomarkers, including inflammatory markers were the strongest

classes of covariates (effect size > 0.2) (**Supplementary Fig. 2c**). However, despite the existence of microbiome variations and their correlation with clinical states, no significant differences were detected for these clinical variables between metacommunity B and C CD patients (**Supplementary Fig. 2d**).

CD- and metacommunity-associated functional traits

We next analyzed the functional changes associated with disease status and differences in microbiome structure. We made pair-wise comparisons after performing functional annotation using the Kyoto Encyclopedia of Genes and Genomes (KEGG) database (KEGG, RRID:SCR_012773). A large number of CD- and metacommunity-related functional shifts were identified at the level of pathways and modules (**Fig. 2a**, **Supplementary Table 5** and **Supplementary Table 6**). We observed consistent changes in CD microbiotas in all within- or between- metacommunity comparisons (in B-CD vs A-CT, C-CD vs A-CT, B-CD vs B-CT, and C-CD vs B-CT) (**Fig. 2a**). The composition of the microbiota of CD patients indicated consistent changes in the potential for carbohydrate utilization compared to the CT counterparts with a decreased abundance of pathways involved in starch and sucrose metabolism, and enrichment of pathways involved in simple carbon metabolism such as fructose, mannose, and galactose in the microbiota of CD patients (**Fig. 2a**). In addition, we observed an enrichment of genes in pathways involved in glyoxylate, dicarboxylate, propanoate and butanoate metabolism as well as in pathways involved in transport of simple sugars (phosphotransferase system) (**Fig. 2a**). Interestingly, the reporter scores of numerous

amino acid metabolic pathways exhibited marked decreases or increases in CD patients compared to CTs, suggesting possible significant changes in the amino acid metabolic profiles (**Fig. 2a**). Of note, the potential for methane metabolism was also diminished in CD patients (**Fig. 2a**). By contrast, microbes in CD patients exhibited enhanced potential for xenobiotic degradation (e.g. of toluene, fluorobenzoate, styrene, benzoate, dioxin, and xylene) and antioxidant defense (e.g. ascorbate, aldarate and glutathione metabolism) (**Fig. 2a**). In parallel, a number of pathways associated with pathogenesis and virulence, including ABC transporters, bacterial secretion system, and general LPS biosynthesis exhibited an incremental enrichment from metacommunity A to C (**Fig. 2a**).

LPS, an inherent component of Gram-negative bacteria, is an endotoxin that can have opposing effects on the immune response [20]. Since pathway and module analyses showed an enrichment of general LPS biosynthesis in the CD microbiome (**Fig. 2a**), we took a novel approach and investigated the capacity amongst all Gram-negative bacteria to produce the pro-inflammatory hexa-acylated LPS as compared to the antagonizing silencing penta-acylated LPS variant [21, 22]. We listed bacteria with a potential for synthesizing each LPS variant (**Supplementary Table 7**) and compared the abundances of these bacteria (**Supplementary Table 8**). The hexa-acylated LPS producing bacteria, *E. coli* and *Morganella morganii* exhibited higher abundance in CD patients from metacommunity C compared to non-CD individuals from metacommunity A (**Supplementary Table 7**). Consistently, compared to metacommunity A (CT), microbes in metacommunity C (CD) tended to produce LPS in a higher hexa- to penta-ratio, suggested by the increase in abundance of bacteria with the hexa- over the penta-acetylated LPS variant (**Fig. 2b**), which

in part may account for an increased inflammatory stimulation of the CD gut.

The abundances of Gram-positive bacteria were reduced in metacommunity C, and in metacommunity B as compared to CTs (**Fig. 2b**). These bacteria make up the largest reservoir for production of SCFAs. SCFAs are not only colonotrophic nutrients but also immunoregulatory molecules [23] that may reduce pro-inflammatory cues within the gut environment. We estimated the abilities of the metacommunities to produce the SCFAs acetic acid, propionic acid and butyric acid. This was done based on the presence of the genes encoding the last enzyme within the respective biosynthetic pathway, thereby providing an alternative method for predicting the capacity for biosynthesis of the bioactive end products than that used in **Fig. 2a**, which was based on presence of genes involved in overall metabolic pathways. Bacteria with a potential to produce SCFAs are listed in **Supplementary Table 7**. Evidently, CD microbiotas, particularly those in metacommunity C, showed a decreased abundance of key genes for SCFA production, including acetic acid, propionic acid and butyric acid, when compared to the CT microbiota in metacommunity A (**Fig. 2c**). Concordantly, the abundance of many SCFA-producing bacteria differed between CT and CD samples (**Supplementary Table 8**). Thus, the gut microbiota in CD patients likely produces a suboptimal amount of SCFAs compared to the healthy state.

Disruption of normal gut microbial ecosystem and bacterial growth rate in CD

The structure of a microbiota is the result of dynamic interactions between community

members. We generated correlation-based microbial interaction networks using the SparCC algorithm (**Fig. 3**, **Supplementary Fig. 3**). Since metacommunity A and C were representative of the typical CT and CD states, respectively, we first compared the microbiome networks of these two groups (**Fig. 3a** and **3b**). The control microbiota in metacommunity A was characterized by a complex network of interactions between different taxa, especially within or between the dominant phyla Bacteroidetes and Firmicutes (**Fig. 3a**). However, the vast majority of these relationships was no longer significant in the CD patients harboring metacommunity C (**Fig. 3b**). Among the strong interactions lost in the gut microbiota of the C-CD group were positive correlations (r>0.5) of *Bacteroides cellulosilyticus* with *Bacteroides thetaiotaomicron* and *Bacteroides* sp., and of *Ruminococcus bromii* with *Eubacterium ventriosum* (**Fig. 3**). Only one new strong correlation was formed between two unidentified taxa in the C-CD group (**Fig. 3**). Thus, the CD microbiota of metacommunity C showed not only alterations in composition, but also reduced interrelationships. In comparison, CT and CD microbiotas from metacommunity B did not differ significantly in terms of network complexity, although numerous inter-taxon relationships were altered (**Supplementary Fig. 3**).

Changes in bacterial growth rate may contribute to alterations in community structures. We calculated the growth rate from the number of sequencing reads covering the replication origin relative to reads covering the replication termination site [24]. Compared to CTs in metacommunity A, the growth rate of many beneficial taxa decreased in metacommunity C, including the SCFA-producing bacteria *Alistipes finegoldii*, *Alistipes shahii*, *Eubacterium*

rectale, *Roseburia intestinalis*, and several *Faecalibacterium prausnitzii* strains (**Fig. 3** and **Supplementary Table 9**). Interestingly, certain pathogenic or opportunistic pathogenic bacteria exhibiting an increased abundance in the C-CD group showed high growth rates (*E. coli*, *Klebsiella pneumoniae*, *Bacteroides fragilis*, and *Streptococcus salivarius*) (**Fig. 3** and **Supplementary Fig. 4** and **Supplementary Table 9**). Thus, differences in growth rate likely contribute to the alterations in the relative abundance of bacteria in CTs and CD patients, since the observed increase or decrease in growth rates largely concurred with their changes in relative abundance in CD samples (**Supplementary Fig. 4**). The reduction of growth rates for most bacteria in the C-CD group may also be an indicator that this metacommunity structure is unlikely to shift towards increased diversity over time without specific intervention.

Limited remodeling of CD microbiota composition by short-term EEN

Fourteen patients in our cohort underwent EEN treatment after baseline sampling and provided fecal samples after two weeks of treatment. We assessed whether short-term EEN was sufficient to alter the microbiome structure in CD patients. For all patients but one (GZCD029, marked by * in **Fig. 4b**), such short time intervention proved insufficient to change their metacommunity identities (**Fig. 4a**), in accord with no significant change in MD-indices ($p = 0.20$, **Fig. 4a** and **Supplementary Table 1**). However, moderate changes occurred as illustrated by the shift in the relative position of microbiomes along the two principal coordinates within pre-identified clusters (**Fig. 4b**).

Despite the limited remodeling of the overall microbiota composition, two-weeks EEN did

induce a variety of functional alterations (**Fig. 4c**, and **Supplementary Table 12** and **Supplementary Table 13**). In a reverse manner to CD-associated shifts, functions such as LPS biosynthesis and bacterial secretion system became less enriched, while starch and sucrose metabolism and flagellar assembly were enhanced after EEN (**Fig. 4c**), suggesting a partial functional recovery. However, certain CD-driven changes, such as functions associated with ribosomes, one carbon folate pool, PTS, and ABC transporters, were exacerbated after two-weeks EEN (**Fig. 4c**), indicating either side effects or temporal disease progression. Nevertheless, short-term EEN did not affect the abundances of LPS- or SCFA-producing bacteria (**Fig 4d**, **e**, **Supplementary Table 10**) nor their growth rates (**Supplementary Table 11**). However, network re-wiring occurred (**Supplementary Fig. 5**). Rather than interacting with Firmicutes, bacteria from Bacteroidetes tended to interact with each other after EEN treatment (**Supplementary Fig. 5**). By contrast, a majority of Firmicutes in patients after EEN treatment presented more inter-dependences with Proteobacteria and unclassified species compared to those before treatment (**Supplementary Fig. 5**). Overall, the CD microbiota appeared relatively stable and refractory to two-week EEN intervention. Future studies will need to determine if a longer intervention period with EEN will result in restoration of normal functional microbiota in CD patients.

Discussion

Comparative metagenomic analysis of fecal samples from CD and heathy controls revealed pronounced global alterations in the fecal microbiota of CD patients, characterized by two distinct CD metacommunities comprising gradually limited bacterial diversity, by functional aberrations towards a pronounced pro-inflammatory phenotype, and by structural derangements of ecosystem networks.

Metacommunities constitute a robust means to distinguish microbiotas with different traits and of distinct natures. Suggested by their signature microbes (the leverage between beneficial bacteria or opportunistic pathogens) and supported by the MD-index, metacommunity A might be representative of the healthy gut, while metacommunity B and C likely represented a moderately imbalanced and a more pro-inflammatory state associated with CD, respectively. Since the commensal microbiota is closely linked to the health of the host, the classification of metacommunities is a novel promising tool to stratify patients based on their microbiome configuration.

Our study identified systematic functional alterations of CD microbiome that reflected the stressful microenvironment of the CD gut and its predisposition to inflammation. In this respect, the decline in the potential for the biosynthesis of all SCFAs, which may modulate the activation of the immune system and temper inflammation [25, 26], and the appearance of microbes producing the pro-inflammatory hexa-acetylated LPS [20] are salient manifestations of the inflammation-prone nature of the CD microbiota. Although LPS has long been established as a pathogen-associated molecular pattern (PAMP) that triggers immune cascades

[20], it was more recently established that only the hexa-acylated LPS variant is able to activate pro-inflammatory cues via TLR4 in humans [22], while the penta-acylated LPS variant acts as an antagonist [21]. Our finding that the CD microbiota of metacommunity C was enriched in microbes producing hexa-acylated LPS is consistent with previous observations of the increased abundance of the *Enterobacteriaceae* family members in CD [1, 4-7], which are known to stimulate inflammation [27]. Together, these changes may severely affect the host immune system, leading to an unchecked inflammatory state in CD. The reduction in the network complexity of the CD microbiota of metacommunity C reinforced the view that a globally disturbed microbial ecosystem may contribute to this disease. The loss of reciprocal and cross-inhibitory relationships may impair the survival of beneficial microbes and create favorable conditions for the blooming of pathogens. Likewise, it appears to limit the growth of many gut bacteria found in healthy individuals. In this regard, reconstruction of the normal ecosystem and not only the mere introduction of a single or several commensal microbes may be needed to curb CD. In the case of EEN, a longer term of treatment may be needed to achieve this goal. Analysis of the fecal microbiota is widely used as a proxy for studying the gut microbiota composition because of the easiness and noninvasive nature of fecal sampling, and has through the years resulted in deepening the understanding of the relationship between the gut microbiota and IBD [1, 28]. However, new avenues of sampling procedures open up for more comprehensive insights into the role played by the intestinal location of microbial species (luminal or mucosal layer attachment to the small and the large intestine) that, in combination with metagenomic sequencing, would allow

for deeper insights into the inter-individual diversity in ecological dysbalance in CD patients in future studies.

Taken together, our metagenome-scale characterization of the CD gut microbiome supports the notion of a shift towards enhanced pro-inflammatory capacity, which is most pronounced in individuals harboring the severe-state metacommunity C. The level of details in this analysis, also encompassing yet unannotated bacteria, may pave the way for elucidating microbial disturbances predictive for CD by enabling the discovery of composite microbial CD biomarkers. In addition, it may allow for the identification of future therapeutic targets based on microbiota signatures, thereby implementing personalized medicine to CD patients based on the individual microbiome composition.

Methods

Study cohort, EEN treatment and sample collection

49 CD patients and 54 healthy controls were enrolled in this study at the Sixth Affiliated Hospital of the Sun Yat-sen University, Guangdong, China. All patients met the diagnostic criteria for CD, according to the Montreal classification system [29]. Patients diagnosed with diabetes, tumor, cardiovascular, kidney, liver, and metabolic diseases were excluded from this study.

Among these participants, 14 CD patients underwent EEN treatment. ENSURE® (Abbott Laboratories, Abbott Park, USA), PEPTISON®, NUTRISON POWDER® (NUTRICIA,

Danone, Netherlands) and FRESUBIN® (Sino-Swed Pharmaceutical Corp. Ltd, China) were used as the standard oral polymeric formulas, and their ingredients are detailed in Supplementary Table 14. Patients chose from these formulas, with 8 patients selecting ENSURE® and the others selecting a mixture of two or more formulas. Formulas were consumed at 30 kcal/kg per day as the sole nutrient source. Patients who adhered to EEN treatment had their lesion healed.

Fecal samples were collected from all participants at baseline (n=103), and from the EEN-treated CD patients after 2 weeks of treatment (n=14), totaling 117 samples. The fecal samples were immediately frozen and stored at -80°C until being processed. DNA extraction was performed according to the protocols described previously [30].

Metagenomic sequencing and assembly

Paired-end metagenomic sequencing was conducted on the Illumina platform (insert size, 350 bp; read length, 100 bp). Quality control was performed and adaptor and host contamination were filtered. Sequencing reads were de novo assembled into contigs with SOAPdenovo v2.04 [31] (SOAPdenovo2, RRID:SCR_014986) as described previously [30].

Co-abundance gene groups identification and functional annotation

Applying the metagenomic species (MGS) clustering method[16], we clustered genes according to their co-variations in abundance across samples. A group of co-abundant genes was identified as a MGS if it contained 700 or more genes. These MGS were subjected to subsequent analysis. Taxonomic assignment of the mapped genes was performed according to the Integrated Microbial Genomes (IMG, v400) database using an in-house pipeline detailed previously[30], with 70% overlap and 65% identity for assignment to phylum, 85% identity to genus, and 95% identity to species. The relative abundance of a co-abundance gene group was calculated from the relative abundance of its genes.

Differentially enriched KO pathways or modules were identified according to their reporter scores[32], which were calculated from the Z-scores of individual KOs.

We assessed the production capacity for the two LPS forms based on the abundances of genes of the entire lipid A biosynthesis pathway, and separated them into penta-acylated LPS producers (harboring all lipid A pathway genes except for LpxM), and pro-inflammatory hexa-acylated LPS producers (all lipid A pathway genes). MGS with no lipid A pathway genes were assigned as Gram-positive bacteria.

Sequences of SCFA-producing enzymes were retrieved as previously described [33]. Genes in the reference gut microbiome gene catalog[8] were identified as these enzymes (best match according to BlastP (BLASTP, RRID:SCR_001010), identity > 35%, score > 60, E<1e-3), and their relative abundances could then be determined accordingly.

α-Diversity and gene count

α-Diversity (within-sample diversity) was calculated on the basis of the gene profile of each sample according to the Shannon index as described previously [30]. The total gene count in each fecal sample was determined as in ref.[34]. Genes with at least one mapped read were considered present.

PERMANOVA of the influence of clinical and lifestyle factors

Permutational multivariate analysis of variance (PERMANOVA) [30]was performed on the gene-abundance profiles of the samples to assess the effect of each of the factors listed in Table 1. We used Bray-Curtis distance and 9,999 permutations in R (3.10, vegan package, R Project for Statistical Computing, RRID:SCR_001905) [35].

Details of updated LEfSe algorithm

Differential abundance analyses were performed using a less stringent LEfSe algorithm to identify feature microbes whose abundances differed at least in one comparison[5]. Metacommunities and subgroups in metacommunity B were included for comparisons. The biomarker relevance was ranked according to bootstrapped (n=30) logarithmic linear discriminant analysis scores of at least 2. The open source R code is available at https://github.com/andriaYG/LDA-EffectSize.

Effect size analysis

24 metadata covariates and their combined effect size when pooled into the broader predefined categories (blood fat, coagulation, inflammation markers, and plasma biomarkers) was estimated with the *bioenv* function in the vegan R package, which selects the combination of covariates with strongest correlation to microbiota variation (Pearson correlation between Gower distances of covariates and microbiome Bray-Curtis dissimilarity, Supplementary Fig. 2A).

Correlation network inferred by phylogenetic marker genes

Eighty-five MGS, which were previously selected via the detection of microbial community clusters through DMM modelling, were subjected to compositionality data analysis using the SparCC algorithm[36]. Taxon–taxon correlation coefficients were estimated as the average of 20 inference iterations with the strength threshold of 0.25. Correlations with the corresponding empirical P values less than 0.01 were retained, which was calculated via a total of 10,000 simulated data sets. This set of iterative procedures was applied separately to data from CTs and CD patients, and to patients' data before and after EEN to infer the correlation values. Correlation coefficients with magnitude of 0.3 or above were selected for visualization in Cytoscape (version 3.3.0, Cytoscape, RRID:SCR_003032).

Availability of supporting data

The raw sequencing data can be found in the EBI database, under the BioProject number PRJEB15371 [15]. The data sets supporting the results of this article are available in the GigaDB repository [37].

List of abbreviations

CD, Crohn's disease; CT, controls; EEN, exclusive enteral nutrition; IBD, inflammatory bowel disease; GI, gastrointestinal; AIEC, adherent-invasive *Escherichia coli*; MAP, *Mycobacterium avium paratuberculosis*; MGS, metagenomics species; LDA, linear discriminant analysis; LEfSe, linear discriminant analysis effect size; MD-index, microbial dysbiosis index; PCoA, principal coordinate analysis; UA, uric acid; KEGG, Kyoto Encyclopedia of Genes and Genomes; LPS, lipopolysaccharide; SCFA, short-chain fatty acid; PAMP, pathogen-associated molecular pattern; IMG, Integrated Microbial Genomes.

Consent for publication

This study was approved by both the institutional review boards at Sixth Affiliated Hospital of Sun Yat-sen University and the BGI ethics committee. All protocols were conducted in compliance with the Declaration of Helsinki and explicit informed consent was obtained from all participants**.**

Competing interests

The authors declare that they have no competing interests

Funding

This research was supported by the National Natural Science Foundation of China (grant No. 81470795 and 81670606), the Shenzhen Municipal Government of China (grant No. JSGG20160229172752028 and JCYJ20160229172757249).

Authors' contributions

All authors read and approved the final manuscript. Q.H., Jian W., Huanming Y., X.X. and X.L. conceived the study. Q.H. participated in the design of the study. L.X., Y.L., L.L, Faming Z., Q.F., Xiaoping L., J.Y., C.L., J.C., and T.Y. carried out the sample collection and preparation. Y.G. and Z.J. participated in sequence assembly, gene mapping and MGS identification. J.M.L. and S.B. performed the analysis of LPS variants. Z.J. generated the SCFA abundance profile. Y.G. carried out the bioinformatics analysis of metacommunities, functions and networks. Y.G., X.Y., L.M, S.B., K.K. and H.J. wrote the manuscript. K.K., S.B. and H.J. supervised project.

Acknowledgements

We gratefully acknowledge colleagues at BGI-Shenzhen for DNA extraction, library construction, sequencing, and discussions.

References

1. Imhann F, Vich Vila A, Bonder MJ, Fu J, Gevers D, Visschedijk MC, Spekhorst LM, Alberts R, Franke L, van Dullemen HM *et al*: **Interplay of host genetics and gut microbiota underlying the onset and clinical presentation of inflammatory bowel disease**. *Gut* 2016.
2. Barnich N, Darfeuille-Michaud A: **Adherent-invasive Escherichia coli and Crohn's disease**. *Curr Opin Gastroenterol* 2007, **23**:16-20.

3. Hermon-Taylor J, Bull TJ, Sheridan JM, Cheng J, Stellakis ML, Sumar N: **Causation of Crohn's disease by Mycobacterium avium subspecies paratuberculosis**. *Can J Gastroenterol* 2000, **14**:521-539.

4. Ricanek P, Lothe SM, Frye SA, Rydning A, Vatn MH, Tonjum T: **Gut bacterial profile in patients newly diagnosed with treatment-naive Crohn's disease**. *Clin Exp Gastroenterol* 2012, **5**:173-186.

5. Gevers D, Kugathasan S, Denson LA, Vazquez-Baeza Y, Van Treuren W, Ren B, Schwager E, Knights D, Song SJ, Yassour M *et al*: **The treatment-naive microbiome in new-onset Crohn's disease**. *Cell Host Microbe* 2014, **15**:382-392.

6. Morgan XC, Tickle TL, Sokol H, Gevers D, Devaney KL, Ward DV, Reyes JA, Shah SA, LeLeiko N, Snapper SB *et al*: **Dysfunction of the intestinal microbiome in inflammatory bowel disease and treatment**. *Genome Biol* 2012, **13**:R79.

7. Thorkildsen LT, Nwosu FC, Avershina E, Ricanek P, Perminow G, Brackmann S, Vatn MH, Rudi K: **Dominant fecal microbiota in newly diagnosed untreated inflammatory bowel disease patients**. *Gastroenterol Res Pract* 2013, **2013**:636785.

8. Li J, Jia H, Cai X, Zhong H, Feng Q, Sunagawa S, Arumugam M, Kultima JR, Prifti E, Nielsen T: **An integrated catalog of reference genes in the human gut microbiome**. *Nature biotechnology* 2014, **32**:834-841.

9. Quince C, Ijaz UZ, Loman N, Eren AM, Saulnier D, Russell J, Haig SJ, Calus ST, Quick J, Barclay A: **Extensive modulation of the fecal metagenome in children with Crohn’s disease during exclusive enteral nutrition**. *The American journal of gastroenterology* 2015, **110**:1718-1729.

10. Torres J, Mehandru S, Colombel JF, Peyrin-Biroulet L: **Crohn's disease**. *Lancet* 2016.

11. Buchman AL: **Side effects of corticosteroid therapy**. *J Clin Gastroenterol* 2001, **33**:289-294.

12. Wall CL, Day AS, Gearry RB: **Use of exclusive enteral nutrition in adults with Crohn's disease: a review**. *World J Gastroenterol* 2013, **19**:7652-7660.

13. Day AS, Lopez RN: **Exclusive enteral nutrition in children with Crohn's disease**. *World J Gastroenterol* 2015, **21**:6809-6816.

14. Kaakoush NO, Day AS, Leach ST, Lemberg DA, Nielsen S, Mitchell HM: **Effect of exclusive enteral nutrition on the microbiota of children with newly diagnosed Crohn's disease**. *Clinical and translational gastroenterology* 2015, **6**:e71.

15. He Q GY, Jie Z, Yu X, Laursen JM, Xiao L, et al.: **The gut microbiome in Crohn's disease and modulation by exclusive enteral nutrition**. In. EMBL-EBI; 2016.

16. Nielsen HB, Almeida M, Juncker AS, Rasmussen S, Li J, Sunagawa S, Plichta DR, Gautier L, Pedersen AG, Le Chatelier E *et al*: **Identification and assembly of genomes and genetic elements in complex metagenomic samples without using reference genomes**. *Nat Biotechnol* 2014, **32**:822-828.

17. Holmes I, Harris K, Quince C: **Dirichlet multinomial mixtures: generative models for microbial metagenomics**. *PLoS One* 2012, **7**:e30126.

18. Segata N, Izard J, Waldron L, Gevers D, Miropolsky L, Garrett WS, Huttenhower C: **Metagenomic biomarker discovery and explanation**. *Genome Biol* 2011, **12**:R60.

19. Atarashi K, Tanoue T, Shima T, Imaoka A, Kuwahara T, Momose Y, Cheng G, Yamasaki S, Saito T, Ohba Y *et al*: **Induction of colonic regulatory T cells by**

indigenous Clostridium species. *Science* 2011, **331**:337-341.

20. Raetz CR, Whitfield C: **Lipopolysaccharide endotoxins**. *Annu Rev Biochem* 2002, **71**:635-700.

21. Park BS, Song DH, Kim HM, Choi B-S, Lee H, Lee J-O: **The structural basis of lipopolysaccharide recognition by the TLR4–MD-2 complex**. *nature* 2009, **458**:1191-1195.

22. Brix S, Eriksen C, Larsen JM, Bisgaard H: **Metagenomic heterogeneity explains dual immune effects of endotoxins**. *Journal of Allergy and Clinical Immunology* 2015, **135**:277.

23. Puertollano E, Kolida S, Yaqoob P: **Biological significance of short-chain fatty acid metabolism by the intestinal microbiome**. *Current opinion in clinical nutrition and metabolic care* 2014, **17**:139-144.

24. Korem T, Zeevi D, Suez J, Weinberger A, Avnit-Sagi T, Pompan-Lotan M, Matot E, Jona G, Harmelin A, Cohen N *et al*: **Growth dynamics of gut microbiota in health and disease inferred from single metagenomic samples**. *Science* 2015, **349**:1101-1106.

25. Furusawa Y, Obata Y, Fukuda S, Endo TA, Nakato G, Takahashi D, Nakanishi Y, Uetake C, Kato K, Kato T *et al*: **Commensal microbe-derived butyrate induces the differentiation of colonic regulatory T cells**. *Nature* 2013, **504**:446-450.

26. Singh N, Gurav A, Sivaprakasam S, Brady E, Padia R, Shi H, Thangaraju M, Prasad PD, Manicassamy S, Munn DH *et al*: **Activation of Gpr109a, receptor for niacin and the commensal metabolite butyrate, suppresses colonic inflammation and carcinogenesis**. *Immunity* 2014, **40**:128-139.

27. Jensen SR, Mirsepasi-Lauridsen HC, Thysen AH, Brynskov J, Krogfelt KA, Petersen AM, Pedersen AE, Brix S: **Distinct inflammatory and cytopathic characteristics of Escherichia coli isolates from inflammatory bowel disease patients**. *International Journal of Medical Microbiology* 2015, **305**:925-936.

28. Round JL, Mazmanian SK: **The gut microbiota shapes intestinal immune responses during health and disease**. *Nature Reviews Immunology* 2009, **9**:313-323.

29. Silverberg MS, Satsangi J, Ahmad T, Arnott IDR, Bernstein CN, Brant SR, Caprilli R, Colombel J-F, Gasche C, Geboes K: **Toward an integrated clinical, molecular and serological classification of inflammatory bowel disease: Report of a Working Party of the 2005 Montreal World Congress of Gastroenterology**. *Canadian Journal of Gastroenterology and Hepatology* 2005, **19**:5A-36A.

30. Qin J, Li Y, Cai Z, Li S, Zhu J, Zhang F, Liang S, Zhang W, Guan Y, Shen D: **A metagenome-wide association study of gut microbiota in type 2 diabetes**. *Nature* 2012, **490**:55-60.

31. Luo R, Liu B, Xie Y, Li Z, Huang W, Yuan J, He G, Chen Y, Pan Q, Liu Y: **SOAPdenovo2: an empirically improved memory-efficient short-read de novo assembler**. *GigaScience* 2012, **1**:1.

32. Patil KR, Nielsen J: **Uncovering transcriptional regulation of metabolism by using metabolic network topology**. *Proceedings of the National Academy of Sciences of the United States of America* 2005, **102**:2685-2689.

33. Claesson MJ, Jeffery IB, Conde S, Power SE, O'Connor EM, Cusack S, Harris HM, Coakley M, Lakshminarayanan B, O'Sullivan O *et al*: **Gut microbiota composition**

correlates with diet and health in the elderly. *Nature* 2012, **488**:178-184.

34. Le Chatelier E, Nielsen T, Qin J, Prifti E, Hildebrand F, Falony G, Almeida M, Arumugam M, Batto J-M, Kennedy S: **Richness of human gut microbiome correlates with metabolic markers**. *Nature* 2013, **500**:541-546.

35. Zapala MA, Schork NJ: **Multivariate regression analysis of distance matrices for testing associations between gene expression patterns and related variables**. *Proceedings of the national academy of sciences* 2006, **103**:19430-19435.

36. Friedman J, Alm EJ: **Inferring correlation networks from genomic survey data**. *PLoS Comput Biol* 2012, **8**:e1002687.

37. He Q GY, Jie Z, Yu X, Laursen JM, Xiao L, Li Y, Li L, Zhang F, Feng Q, Li X, Yu J, Liu C, Lan P, Yan T, Liu X, Xu X, Yang H, Wang J, Madsen L, Brix S, Wang J, Kristiansen K, Jia H. : **Supporting data for "Two distinct metacommunities characterize the gut microbiota in Crohn's disease patients"** In*.* GigaScience Database. 2017. http://dx.doi.org/10.5524/100317.

Figure legends

Figure 1. Clustering of gut microbiota into metacommunities associated with CD. (a) Heatmap of signature microbes for three metacommunities determined by the DMM model. Rows correspond to 85 discriminative MGS, with hierarchical clustering by their relative abundances. Taxonomic annotations of these MGS are indicated at the right and colored by phylum. Each column corresponds to one sample. The disease status (the first horizontal bar) and metacommunity membership (the second horizontal bar) of samples are indicated by color at the top, and MD index for each sample is represented by gray scale (the third horizontal bar). **(b)** PCoA of the 85 MGS based on Jensen-Shannon distance (JSD). Colors indicate metacommunity memberships, and shapes (triangle or round) denote disease states (CT or CD).

Figure 2. Functional alterations of the gut microbiota in CD. (a) Heatmap and hierarchical clustering of KEGG pathways that are differentially enriched between the microbiota groups identified in Fig 1a. Color scale represents reporter score, and only KEGG pathways with a reporter score greater than 1.9 are shown. **(b)** Relative abundances of Gram-negative MGS (the first left panel), Gram-positive MGS (the second left panel), penta-acylated LPS producing MGS (the middle panel), hexa-acylated LPS producing MGS (the second last panel), and the ratio of hexa- to penta-acylated LPS producing MGS (the last panel) across different groups. The value of relative abundance was log-transformed. **(c)** Relative abundances of genes encoding key enzymes for the biosynthesis of different SCFAs across

different microbiota groups. Carbon monoxide dehydrogenase and acetyl CoA synthase complex are crucial for acetic acid production; propionyl-CoA transferase and propionyl-CoA/succinyl-CoA transferase are responsible for propionate acid synthesis; butyryl CoA transferase accounts for butyric acid generation. Their relative abundances were log-transformed. **(b,c)** Statistical comparison by Wilcoxon test followed by a Benjamini-Hochberg correction for significance level; *q<0.2; **q<0.1; ***q<0.05; ****q<0.001.

Figure 3. Reconstruction of microbial interaction networks by CD. Co-occurrence (blue) relationships and co-exclusion (red) between taxa were estimated by SparCC algorithm, and correlation networks were compared between non-CD samples from metacommunity A (**a**, A-CT) and CD samples from metacommunity C (**b**, C-CD). Only relationships with coefficients above 0.3 are visualized, and the thickness of lines denotes strength of correlation as indicated in the legend. Node size represents mean taxon abundance in networks, and node color represents the growth rate of each species (grey indicates no detection). Taxa of the same bacterial phylum are encircled by dashed lines.

Figure 4. Moderate modification of CD microbiota by EEN treatment. (a) Gut MGS from CD patients (n=14) before and after 14 days of EEN were clustered into metacommunities and visualized as a heatmap representing the 85 discriminative MGS (as in Fig. 1a). Each column corresponds to one sample. **(b)** PCoA of pre- and post-EEN CD microbiota based on Jensen-Shannon distance (JSD). Arrows indicate the shift of position along the first two

principal coordinates pre- to post-EEN treatment. The sample whose metacommunity identity changed after EEN treatment is marked with an asterisk (GZCD029). **(c)** Heatmap and hierarchical clustering KEGG pathways that were enriched or decreased in post- versus pre-EEN. Color scale represents reporter score, and only KEGG pathways with a reporter score greater than 1.9 are shown. **(d)** Log_{10} relative abundances of Gram-negative MGS (the first left panel), Gram-positive MGS (the second left panel), penta-acylated LPS producing MGS (the middle panel), hexa-acylated LPS producing MGS (the second last panel), and the ratio of hexa- to penta-acylated LPS producing MGS (the last panel) in pre- versus post-EEN. **(e)** Log_{10} relative abundances of genes encoding key enzymes for the biosynthesis of different SCFAs in pre- versus post-EEN, as calculated in Figure 2c. **(d,e)** Statistical comparison by Wilcoxon test followed by a Benjamini-Hochberg correction for significance level showed no changes between groups.

Figure1

Click here to download Figure figure 1.v3.pdf

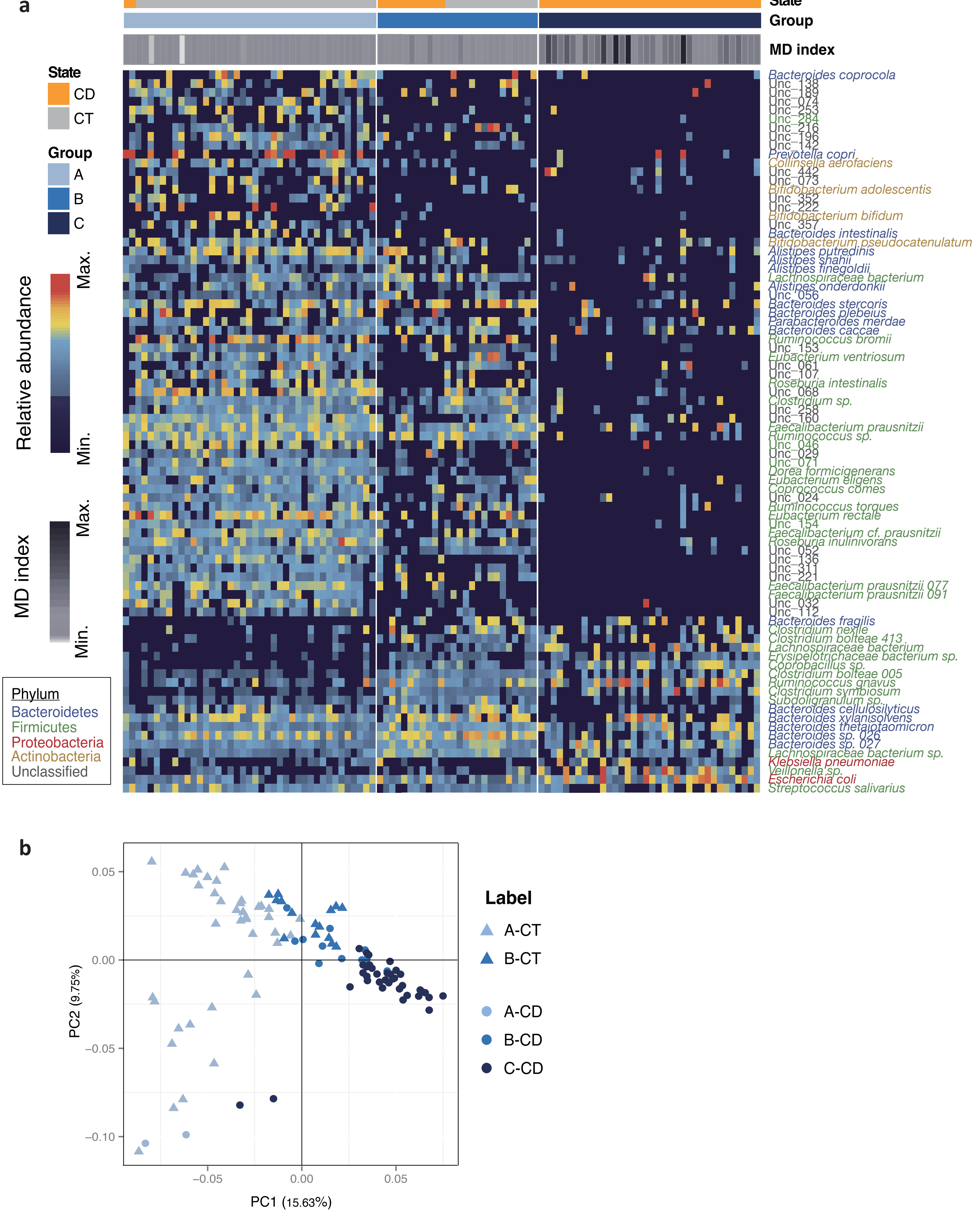

Figure2

Click here to download Figure figure 2.v3.pdf

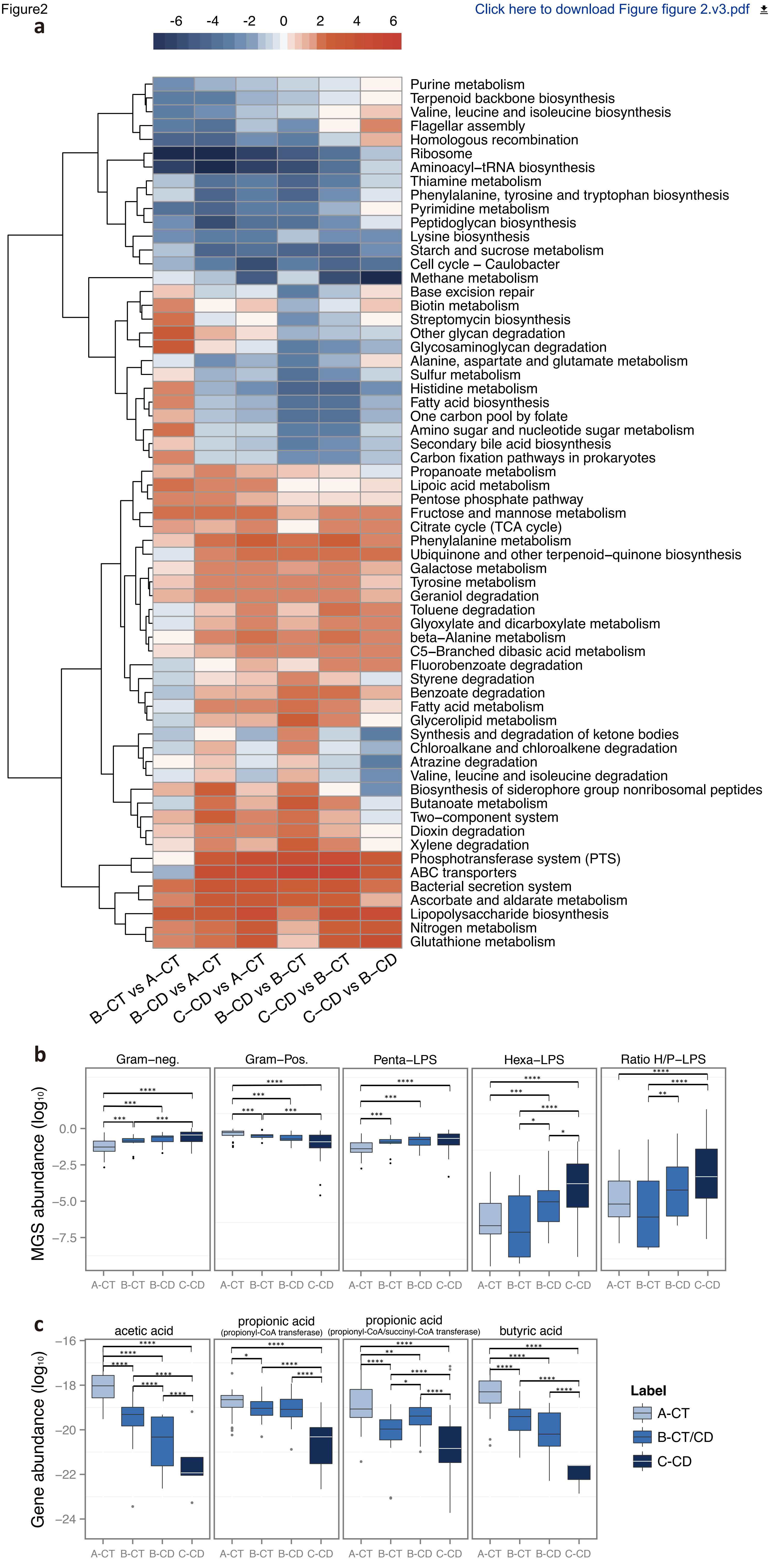

Figure3

Click here to download Figure figure 3.v3.pdf

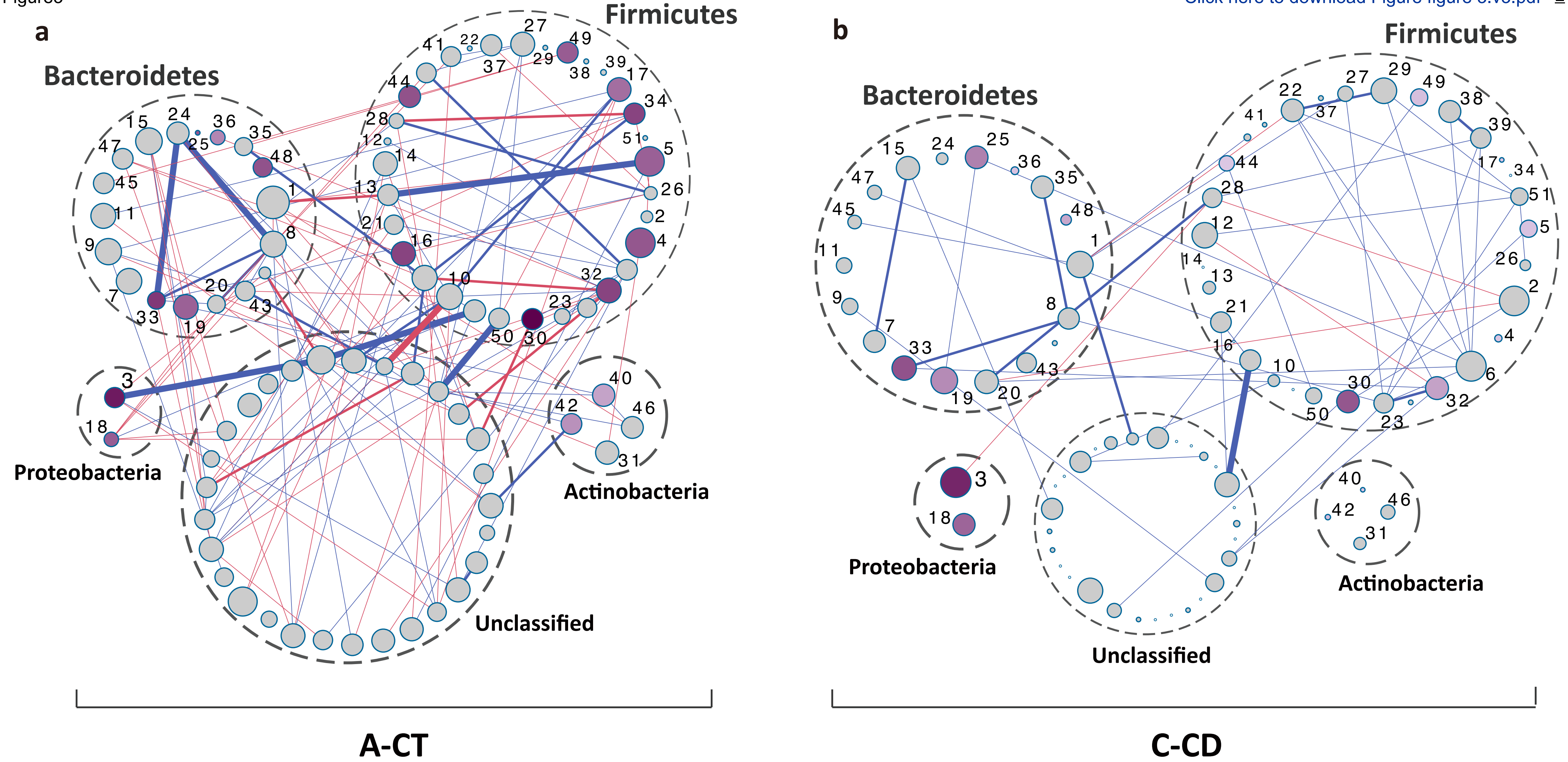

1. *Prevotella copri*
2. *Veillonella sp.*
3. *Escherichia coli*
4. *Eubacterium rectale*
5. *Ruminococcus bromii*
6. *Ruminococcus gnavus*
7. *Bacteroides stercoris*
8. *Bacteroides sp. 026*
9. *Alistipes putredinis*
10. *Roseburia inulinivorans*
11. *Bacteroides coprocola*
12. *Lachnospiraceae bacterium 100*
13. *Eubacterium ventriosum*
14. *Faecalibacterium cf. prausnitzii*
15. *Bacteroides plebeius*
16. *Faecalibacterium prausnitzii 077*
17. *Roseburia intestinalis*
18. *Klebsiella pneumoniae*
19. *Bacteroides xylanisolvens*
20. *Bacteroides sp. 027*
21. *Lachnospiraceae bacterium 065*
22. *Coprobacillus sp.*
23. *Clostridium nexile*
24. *Bacteroides cellulosilyticus*
25. *Bacteroides fragilis*
26. *Subdoligranulum sp.*
27. *Ruminococcus sp.*
28. *Lachnospiraceae bacterium sp.*
29. *Clostridium symbiosum*
30. *Streptococcus salivarius*
31. *Collinsella aerofaciens*
32. *Faecalibacterium prausnitzii 094*
33. *Bacteroides thetaiotaomicron*
34. *Faecalibacterium prausnitzii 091*
35. *Bacteroides caccae*
36. *Alistipes finegoldii*
37. *Coprococcus comes*
38. *Clostridium bolteae 413*
39. *Clostridium bolteae 005*
40. *Bifidobacterium bifidum*
41. *Dorea formicigenerans*
42. *Bifidobacterium adolescentis*
43. *Alistipes onderdonkii*
44. *Eubacterium eligens*
45. *Bacteroides intestinalis*
46. *Bifidobacterium pseudocatenulatum*
47. *Parabacteroides merdae*
48. *Alistipes shahii*
49. *Ruminococcus torques*
50. *Clostridium sp.*
51. *Erysipelotrichaceae bacterium sp.*

Growth rate

Min. Max.

Co-occurring

$r > 0.3$

$r > 0.4$

$r > 0.5$

Co-excluding

$r < -0.3$

$r < -0.4$

$r < -0.5$

Figure4

Click here to download Figure figure 4.v4.pdf

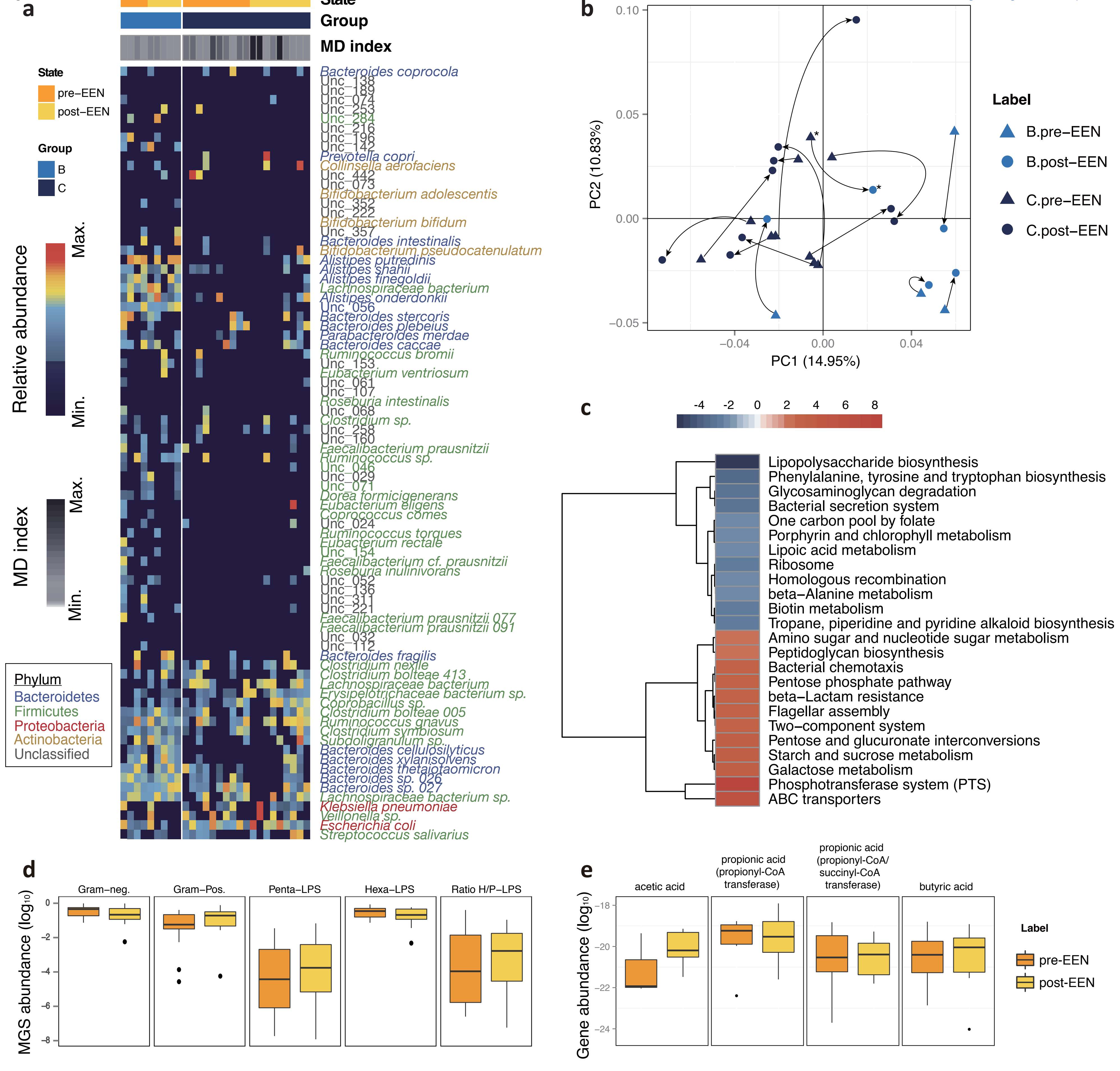

Click here to access/download
Supplementary Material
Table & Supplementory Table.CD.xlsx

Click here to access/download
Supplementary Material
SI-CD-paper-gigascience.docx
