# Supplementary material for: Two distinct metacommunities characterize the gut microbiota in Crohn's disease patients
Source: Gigascience. 2017 Jun 26;6(7):1–11. doi: 10.1093/gigascience/gix050 (PMC5624284; doi:10.1093/gigascience/gix050)
Supplement: SI-CD-paper-gigascience.docx [file gix050_SI-CD-paper-gigascience.docx]

**Supplementary Information**

for

**The gut microbiome in Crohn's disease and modulation by exclusive enteral nutrition**

# Supplementary Figures

****Supplementary Figure 1. Gene counts and microbial diversity of non-CD and CD microbiota and model fit of Dirichlet mixtures.

(a) Rarefaction curve of microbial gene counts in non-CD (CT) and CD samples. (b) Shannon index (α-diversity) at gene level for CT and CD microbiota. Wilcoxon test was performed. ****p<0.001. (c) Evaluation of the model fit for increasing number of Dirichlet mixture components using the Naïve Bayes to the negative log model evidence, where x-axis represents cluster number and y-axis represents negative log posterior (model fit).

**
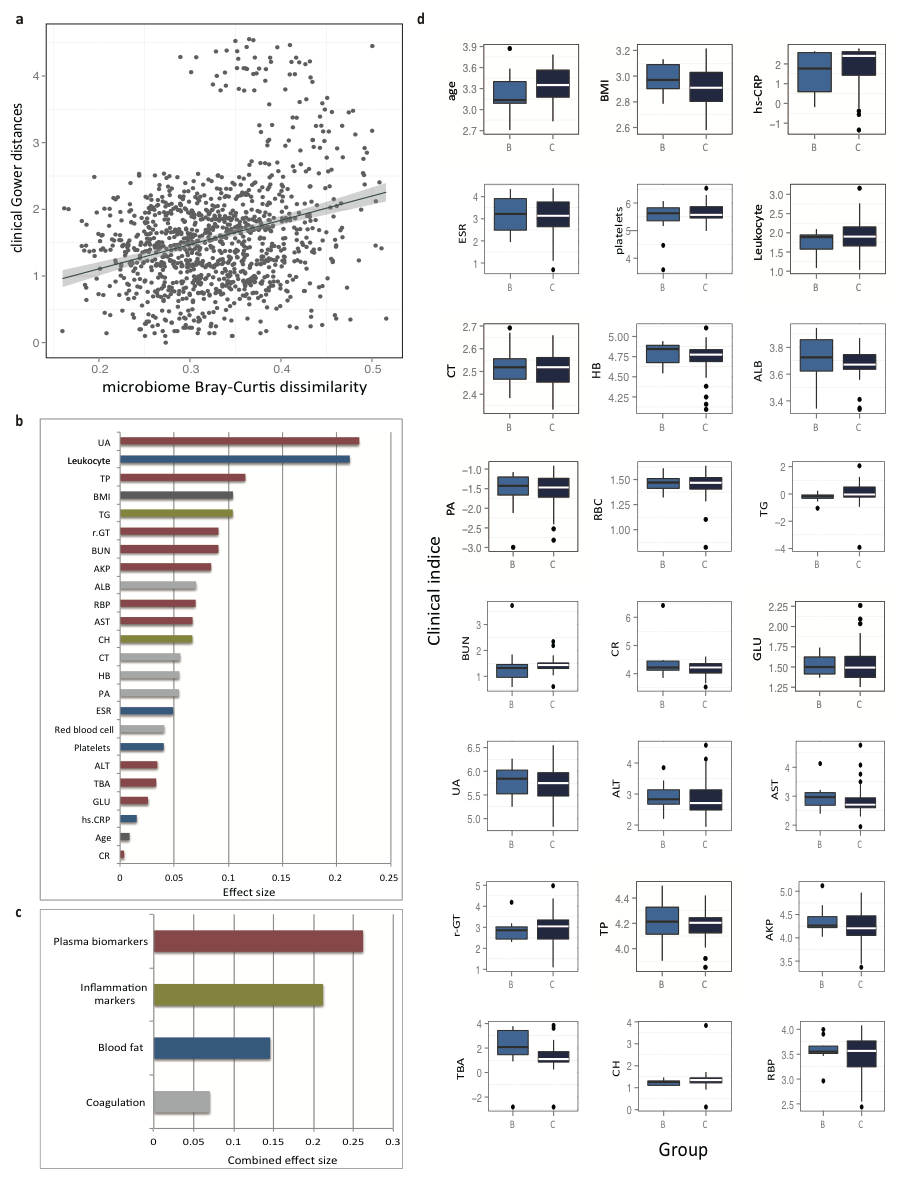
**

**Supplementary Figure 2. Effect sizes of clinical covariates on microbiome composition. (a)** An example illustrating the way by which effect size was calculated, through Pearson correlation between Gower distances of covariate (y-axis) and microbiome Bray-Curtis dissimilarity (x-axis). Effect size equals to Pearson correlation coefficient. **(b, c)** Effect sizes of blood test covariates on microbiome composition individually **(b)** and when categorized into groups **(c).** Combined effect sizes were determined by pooling effect sizes of all indices within the same category (of the same color in supplementary **Fig. 2b**) with covariate distance-based selection. **(d)** Comparison of clinical covariates between CD patients from metacommunity B and C. Statistical comparison by Wilcoxon test followed by a Benjamini-Hochberg correction for significance level.

**Supplementary Figure 3. Microbial interaction networks in non-CD and CD microbiota from metacommunity B.** Co-occurrence (blue) relationships and co-exclusion (red) between taxa were estimated by SparCC algorithm, correlation networks were compared between non-CD samples (**a,** B-CT) and CD samples (**b,** B-CD) from metacommunity B. Only relationship coefficients above 0.3 are visualized, and the thickness of lines denotes strength of correlation as indicated in the legend. Node size represents mean taxon abundance in networks, and node color represents the growth rate of each species (grey indicates no detection). Taxa of the same bacterial phylum are encircled by dashed lines.

**Supplementary Figure 4. Consistency between microbial relative abundance and growth rate. (a, b)** Percentages of MGS (q<0.2) in microbiota that differed in abundance between non-CD samples from metacommunity A (**a,** A-CT) and CD samples from metacommunity C (**b,** C-CD), calculated based on relative abundances. **(c, d)** Juxtaposition of the differences in bacterial relative abundance **(c)** and growth rate **(d)** between A-CT and C-CD. Bar represents z-score, which was calculated by using the cumulative distribution of p value in Wilcoxon test. Positive z-score indicates enrichment in C-CD, while negative z-score indicates enrichment in A-CT.

**Supplementary Figure 5. Microbial interaction networks of CD microbiota at pre- and post-EEN treatment.** Co-occurrence (blue) relationships and co-exclusion (red) between taxa were estimated by SparCC algorithm, correlation networks were compared between CD microbiota at pre- (**a**, pre-EEN) and post-EEN treatment (**b**, post-EEN). Only relationships with coefficients above 0.3 are visualized, and the thickness of lines denotes strength of correlation. Node size represents mean taxon abundance in networks, and node color represents the growth rate of each species (grey indicates no detection). Taxa of the same bacterial phylum are encircled by dashed lines.

Supplementary Tables

**Supplementary Table 1. Clinical characteristics of participants and the dysbiosis index for their gut micorbiome.** This table includes non-CD controls (n=54), CD patients (n=49) without/before EEN treatment and a subset of CD patients profiled 2 weeks after EEN treatment (n=14).

Supplementary Table 2. **MGS of the IBD cohort.** Clusters containing > 700 genes were annotated according to available bacteria and archaea genomes, as was described previously (Qin et al. 2012).

Supplementary Table 3. **Association between metacommunity and CD status.** *P*-values from Fisher’s exact tests were adjusted by Benjamini-Hochberg step-up procedure.

Supplementary Table 4. **Results of differential abundance analysis on signature MGS for metacommunities.** An adapted version of the linear discriminant analysis (LDA) effect size (LEfSe) method were applied for selecting differential MGS. Those with a LDA score over 2 were visualized in **Fig. 1** and Supplementary Table 4.

**Supplementary Table 5. Summary of differential abundance analysis on KEGG pathways between subgroups.** Differentially enriched KO pathways were identified according to their reporter scores.

**Supplementary Table 6. Summary of differential abundance analysis on KEGG modules between subgroups.** Differentially enriched KO pathways were identified according to their reporter scores.

**Supplementary Table 7. A list of LPS/SCFA-producing bacteria that had differentially abundance/growth rate between subgroups (q<0.2).** Statistical comparison by Wilcoxon test followed by a Benjamini-Hochberg correction for significance level.

Supplementary Table 8. **Results of Wilcoxon Test on the relative abundance of all MGS between sub-groups.** A Benjamini-Hochberg correction was applied for significance level.

Supplementary Table 9. **Results of Wilcoxon Test on the growth rate of all MGS between sub-groups.** A Benjamini-Hochberg correction was applied for significance level.

Supplementary Table 10. **Results of Wilcoxon Test on the relative abundance of all MGS in pre- versus post-EEN CD samples.** A Benjamini-Hochberg correction was applied for significance level.

Supplementary Table 11. **Results of Wilcoxon Test on the growth rate of all MGS in pre- versus post-EEN CD samples.** A Benjamini-Hochberg correction was applied for significance level.

Supplementary Table 12. **Summary of differential abundance analyses on KEGG pathways in pre-EEN and post-EEN CD samples.** Differentially enriched KO pathways were identified according to their reporter scores.

Supplementary Table 13. **Summary of differential abundance analyses on KEGG modules in pre-EEN and post-EEN CD samples.** Differentially enriched KO modules were identified according to their reporter scores.

Supplementary Table 14. **Detailed formula of 4 nutrition powder applied in this study.**
