# Supplementary material for: Two distinct metacommunities characterize the gut microbiota in Crohn's disease patients
Source: Gigascience. 2017 Jun 26;6(7):1–11. doi: 10.1093/gigascience/gix050 (PMC5624284; doi:10.1093/gigascience/gix050)
Supplement: Response_to_Reviewer_Comments_Original_Submission.pdf [file gix050_Response_to_Reviewer_Comments_Original_Submission.pdf]

Author's Response To Reviewer Comments

We thank the Reviewers for their helpful comments. The manuscript has now been revised to address all these concerns.

Reviewer #1: The manuscript by He et al presents shotgun metagenomics data and analysis on healthy controls and CD patients before and after exclusive enteral nutrition. The results presented are interesting and the authors are to be commended for such deep sequencing (~55 million reads) of their samples. However, I do have several comments that I believe need to be addressed.

1. I am quite surprised at the level of unclassified sequences at the phylum level in Figure 1 and Figure 3. The authors data (in Figure 3 at least) seems to focus on four phyla. Some recent papers have detected up to 21 phyla in fecal samples using shotgun metagenomics (Bonder et al 2016 Nature Genetics). Could this be a classification issue?

We thank the Reviewer for this question. It is worth noting that only about 10%-20% of bacterial species are shared by the general population, and most commensal bacteria are hitherto uncharacterized. Therefore, the total number of taxa identified in a cohort depends on the sample size and population characteristics. Conceivably, pooling microbiome samples from a large cohort with heterogeneous traits (e.g. Bonder et al. 2016 Nature Genetics) leads to the identification of a large variety of phyla, but most of them may be individual-specific. The relatively small number of phyla identified in our cohort is possibly in part due to the uniqueness of our cohort (patients with Crohn's disease have a low diversity of gut microbiome). Besides, since no all-inclusive bacterial genome database exists and the commonly used ones (e.g. IMG, NCBI and Metaphlan2) have their own strength and weaknesses, phylogenetic annotation is also dependent on the database used (IMG was mainly used in our study). The advantage of clustering microbial entities by MGS lies in its identification of undocumented species.

We have compared the number of bacterial taxa (at phylum and species level) identified by Metaphlan2 and those based on the MGS method used in our study (Table i). The list of phyla is also shown in Table ii. We note that comparable numbers of phyla and species are identified by these two approaches, both before and after eliminating the rarely occurring taxa. The additional phylum annotated by Metaphlan2 but not by MGS (Synergistetes) contains only one species that marginally passed our occurrence cutoff (at 10.26% occurrence rate). Thus, the MGS method is no less robust than Metaphlan2 in terms of taxonomic coverage. More importantly, we are able to detect the key CD-associated compositional (metacommunities) and functional alterations (e.g. the production of LPS and its variants) based on our clustered MGSs, so taxonomic coverage is not a major issue for our study.

Table i Number of bacterial taxa identified by Metaphlan2 and by MGS-based method. (see attach file GIGA-D-17-00073 pbp response.docx)

Table ii The list of phyla identified by Metaphlan2 and by MGS-based method. Metaphlan2.(see attach file GIGA-D-17-00073 pbp response.docx)

2. A marker based tool such as Metaphlan2 would provide taxonomic classification to be compared to the authors classifications. The authors can also perform hierarchal clustering through Metaphlan2 to confirm their metacommunities.

We thank the Reviewer for this suggestion. As mentioned in the response above, Metaphlan2 and MGS identify similar numbers of species with a small taxonomic overlap in our samples (Table i). However, when using the microbial profiles attained by Metaphlan2 as the input data to compare non-CD and CD microbiota, discrimination between these two groups is not satisfactory: non-CD and CD samples cannot be well separated in Jensen-Shannon distance-based PCoA (the following figure below, upper panel), in contrast to the ordination result based on MGS profiles (Fig. 1b). It suggests that compared to Metaphlan2 microbial profiles, our set of MGS contain more bacteria that are specifically related to non-CD individuals, including some so-far unannotated species, and on the basis of these bacteria, we are more likely to pinpoint the CD-associated microbiota compositional shift. Nevertheless, microbiota compositions produced by Metaphlan2 still show high similarity within the metacommunities that are categorized based on MGS in our study, demonstrated by the proximity of samples within each metacommunity on the first two coordinates of PCoA (the following figure, lower panel), so metacommunity modeling of MGS composition is comparable to bacterial profiling by other methods, at least for Metaphlan2.
With these results in mind, we concluded that hierarchal clustering through Metaphlan2 was not warranted, the more so considering the shortcomings of hierarchal clustering for metacommunity classification, because this protocol does not permit an autonomously search for the optimal number of groups.

Jensen-Shannon distance-based PCoA was performed on the microbial profiles derived by Metaphlan 2. Control(CT) and CD samples (CD) were indicated by shape. Metacommunity membership (clustered based on MGS profiles, as specified in the manuscript) was represented by color (the lower panel).

3. There seems to be an absence of microbial eukaryotic and viral annotated reads. Can the authors address this?

We thank the Reviewer for this question. To unequivocally study the commensal viruses present in the gut, special isolation and nucleotide extraction method are required to enrich for viral particles in the fecal samples, and reverse transcription is needed prior to the sequencing of RNA viruses. The DNA extraction protocol in our study is not applicable to virus investigation. We have attempted to annotate the eukaryotes that are present in the gut of our cohort with Metaphlan2. No microbial eukaryotes were identified in our individuals at the cutoff of 10% occurrence.

4. Based on all the patient metadata available, I believe the authors should apply MaAsLin in addition to their application of LEfSe on the data.

We thank the Reviewer for this suggestion. Apart from the disease status of CD, age, gender, and BMI are the other variables that affect gut microbiome (Table 1). However, we are very cautious

against adjusting for BMI in our microbiome study, because it is likely an "outcome" of CD and certain CD-associated differences may be missing if adjusting for this variable. In terms of the other two variables, the two groups (control and CD) do not differ greatly in age, and they are only slightly imbalanced in gender. After adjusting age and gender using MaAsLin, only 6 out of 85 MGS are no longer different in their relative abundance between control and CD groups (please see the attached excel sheet "GIGA-D-17-00073 maaslin"), so we do not think these two confounders will significantly bias our conclusions.

5. The authors refer to ~55 million reads per sample. This level of depth can result in strain level analysis and perhaps even partial genomic assembly of more abundant bacterial strains. Functional inferences can be obtained from much less reads per sample. Can the authors address this?

We thank the Reviewer for this suggestion. In the present study, we are interested in the global microbiota compositional shifts and functional deviations associated with CD. To date, there is no consensus on the definition of a strain. Moreover, in order to uncover the intra-strain genomic differences between groups, one or several species of interest that may have strain variations should be pinpointed in the first place, which is like looking for a needle in a haystack in the metagenomics data.

6. Most studies utilizing EEN as a therapy do this over a long period of time (6-8 weeks). Can the authors justify limiting the EEN to 2 weeks? Could this explain the limited effects the authors observed?

We thank the Reviewer for these questions. We re-sampled the patients after two-week EEN due to either loss of follow-up or patients' non-adherence to EEN after two weeks of treatment. Still, modification of microbiota functions can be detected after two weeks of EEN. We are convinced that these preliminary findings are of interest to scientists and clinicians in this field.

7. Citations of work related to CD and EEN are missing. There are several studies on this topic that have shown microbiome changes, including one study that has utilized shotgun metagenomics. The authors should also correct their statement in the introduction related to this. Please refer to the following paper (Quince C et al 2015 Am J Gastroenterol).

We thank the Reviewer for this suggestion. This work has now been cited in the revised manuscript (line 68-73; line 86-87).

8. The authors have provided two titles, one during submission and one at the start of the manuscript. I assume the submission title is the relevant one?

We apologize for the confusion. The title submitted for publication is "Two distinct metacommunities characterize the gut microbiota in Crohn's disease patients".

Reviewer #2 (reviewed your original submission and has re-reviewed and looked at the comments you addressed): The authors have addressed all the previously raised points and the manuscript has been improved respect to the precedent version. Minor language revisions will be

advisable. However, the manuscript is acceptable for publication.

We thank the Reviewer for these positive comments.
