# Supplementary material for: Two distinct metacommunities characterize the gut microbiota in Crohn's disease patients
Source: Gigascience. 2017 Jun 26;6(7):1–11. doi: 10.1093/gigascience/gix050 (PMC5624284; doi:10.1093/gigascience/gix050)
Supplement: Response_to_Reviewer_Comments_Revision_1.pdf [file gix050_Response_to_Reviewer_Comments_Revision_1.pdf]

Author's Response To Reviewer Comments

1) Please cite acccession numbers or project numbers for the raw data that you say is deposited in the EBI database. The EBI database then needs to be added as a reference in the references list then only the Reference number cited in the main text, after the accession/ project numbers. See my comments.

Thanks for pointing this out. We have inserted the acccession numbers for raw data and added EBI database as a reference in the references list.

2) We have added in RRIDs to make your manuscript more reproducible- please do not delete this.

Sure, these RRIDs are remained in manuscript. Many thanks.

*the DOI Citation of GigaDB is not available right now, so we did not complete the "Available of supporting data" part.
