# Supplementary material for: Two distinct metacommunities characterize the gut microbiota in Crohn's disease patients
Source: Gigascience. 2017 Jun 26;6(7):1–11. doi: 10.1093/gigascience/gix050 (PMC5624284; doi:10.1093/gigascience/gix050)
Supplement: Reviewer_1_Report_(Original_Submission).pdf [file gix050_Reviewer_1_Report_(Original_Submission).pdf]

Reviewer Report

Title: Two distinct metacommunities characterize the gut microbiota in Crohn's disease patients

Version: Original Submission **Date:** 5/20/2017

Reviewer name: Nadeem Kaakoush

Reviewer Comments to Author:

The manuscript by He et al presents shotgun metagenomics data and analysis on healthy controls and CD patients before and after exclusive enteral nutrition. The results presented are interesting and the authors are to be commended for such deep sequencing (~55 million reads) of their samples. However, I do have several comments that I believe need to be addressed.

1. I am quite surprised at the level of unclassified sequences at the phylum level in Figure 1 and Figure 3. The authors data (in Figure 3 at least) seems to focus on four phyla. Some recent papers have detected up to 21 phyla in fecal samples using shotgun metagenomics (Bonder et al 2016 Nature Genetics). Could this be a classification issue?

2. A marker based tool such as Metaphlan2 would provide taxonomic classification to be compared to the authors classifications. The authors can also perform hierarchal clustering through Metaphlan2 to confirm their metacommunities.

3. There seems to be an absence of microbial eukaryotic and viral annotated reads. Can the authors address this?

4. Based on all the patient metadata available, I believe the authors should apply MaAsLin in addition to their application of LEfSe on the data.

5. The authors refer to ~55 million reads per sample. This level of depth can result in strain level analysis and perhaps even partial genomic assembly of more abundant bacterial strains. Functional inferences can be obtained from much less reads per sample. Can the authors address this?

6. Most studies utilizing EEN as a therapy do this over a long period of time (6-8 weeks). Can the authors justify limiting the EEN to 2 weeks? Could this explain the limited effects the authors observed?

7. Citations of work related to CD and EEN are missing. There are several studies on this topic that have shown microbiome changes, including one study that has utilized shotgun metagenomics. The authors should also correct their statement in the introduction related to this.

Please refer to the following paper (Quince C et al 2015 Am J Gastroenterol).

8. The authors have provided two titles, one during submission and one at the start of the manuscript. I assume the submission title is the relevant one?

Methods

Are the methods appropriate to the aims of the study, are they well described, and are necessary controls included? Yes

Conclusions

Are the conclusions adequately supported by the data shown? Yes

Reporting Standards

Does the manuscript adhere to the journal's guidelines on minimum standards of reporting? YesChoose an item.

Statistics

Are you able to assess all statistics in the manuscript, including the appropriateness of statistical tests used? Yes, and I have assessed the statistics in my report.

Quality of Written English

Please indicate the quality of language in the manuscript: Acceptable

Declaration of Competing Interests

Please complete a declaration of competing interests, considering the following questions:

- Have you in the past five years received reimbursements, fees, funding, or salary from an organisation that may in any way gain or lose financially from the publication of this manuscript, either now or in the future?
- Do you hold any stocks or shares in an organisation that may in any way gain or lose financially from the publication of this manuscript, either now or in the future?
- Do you hold or are you currently applying for any patents relating to the content of the manuscript?
- Have you received reimbursements, fees, funding, or salary from an organization that holds or has applied for patents relating to the content of the manuscript?
- Do you have any other financial competing interests?
- Do you have any non-financial competing interests in relation to this paper?

If you can answer no to all of the above, write 'I declare that I have no competing interests' below. If your reply is yes to any, please give details below.

I declare that I have no competing interests

I agree to the open peer review policy of the journal. I understand that my name will be included on my report to the authors and, if the manuscript is accepted for publication, my named report including any attachments I upload will be posted on the website along with the authors' responses. I agree for my report to be made available under an Open Access Creative Commons CC-BY license (http://creativecommons.org/licenses/by/4.0/). I understand that any comments which I do not wish to be included in my named report can be included as confidential comments to the editors, which will not be published.

I agree to the open peer review policy of the journal

To further support our reviewers, we have joined with Publons, where you can gain additional credit to further highlight your hard work (see: https://publons.com/journal/530/gigascience). On publication of this paper, your review will be automatically added to Publons, you can then choose whether or not to claim your Publons credit. I understand this statement.

Yes
